# First intraindividual comparison of contrast-enhanced MRI, FET- and DOTATOC- PET in patients with intracranial meningiomas

**DOI:** 10.1186/s13014-017-0913-x

**Published:** 2017-11-06

**Authors:** Jan Oliver Dittmar, Clemens Kratochwil, Anne Dittmar, Thomas Welzel, Daniel Habermehl, Stefan Rieken, Frederik L. Giesel, Uwe Haberkorn, Jürgen Debus, Stephanie E. Combs

**Affiliations:** 1 Department of Radiation Oncology, Klinikum rechts der Isar, Technische Universität München (TUM), Ismaninger Str. 22, 81675 Munich, Germany; 2 Department of Radiation Oncology, University Hospital of Heidelberg, Im Neuenheimer Feld 400, 69120 Heidelberg, Germany; 3 Department of Nuclear Medicine, University Hospital of Heidelberg, Im Neuenheimer Feld 400, 69120 Heidelberg, Germany; 4 Institute for Innovative Radiotherapy (iRT), Department of Radiation Sciences (DRS), Helmholtz Zentrum München, 85764 Oberschleißheim, Germany; 5 Deutsches Konsortium für Translationale Krebsforschung (DKTK), Partner Site Munich, Munich, Germany

**Keywords:** Meningioma, PET, DOTATOC, FET, Radiation therapy

## Abstract

**Background:**

For irradiation treatment planning of meningiomas the use of PET-scans is well established. The most frequently used tracers are either based on amino acids or the somatostatin receptor ligand DOTATOC. Since up to now no inter-institutionally accepted standard PET-tracer has been defined, the aim of this study was to evaluate the influence of these different types of PET-tracers on the GTV-definition.

**Methods:**

Twenty-one patients suffering from intracranial meningiomas underwent CT, MRI, FET- and DOTATOC-PET. First, tumour extension was delineated after image-fusion of CT and MRI (GTV_CT/MRI_). Then distinct GTVs based either on FET- or DOTATOC-PET were contoured and compared with each other as well with GTV_CT/MRI_.

**Results:**

Every tumour showed typical enhancement of DOTATOC, but two meningiomas remained FET-negative. The mean relative overlap volume of GTV_FET_ and GTV_DOTATOC_ was only 41.9% and there was a significantly stronger correlation between GTV_CT/MRI_ and GTV_DOTATOC_ than between GTV_CT/MRI_ and GTV_FET_ **.**

**Conclusions:**

Further investigations are necessary to clarify the minor conformity of DOTATOC- and FET-PET in meningiomas. Because of the receptor targeting, DOTATOC is known to be more specific for meningiomas and will remain the standard in our institution with the known limitation in areas nearby the pituitary gland.

## Background

Over the last decades radiation therapy (RT) has been established as a successful, safe and effective treatment of intracranial meningiomas. Because of their mostly benign character, a highly conformal RT is essential to avoid radiation-induced side effects. Better and better high precision RT techniques have been developed in the past like Fractionated Stereotactic Radiotherapy, Intensity Modulated Radiotherapy, Radiosurgery as well as Particle Therapy with protons or carbon ions [1–5], resulting in high control rates with very low rates of side effects [1, 6–10].

However the more precise radiation techniques become, the more an accurate delineation of the gross tumour volume (GTV) is essential. Distinguishing between tumour and surrounding normal tissue can be difficult even by combining computed tomography (CT) and magnetic resonance imaging (MRI) e.g. for evaluation of bony involvement or the dural tail [11, 12]. Furthermore, strong enhancement of contrast fluid in CT- and MRI-scans is seen both in meningioma cells and neighbouring normal tissue like the cavernous or sagittal sinus. Therefore a trimodal image fusion using CT, MRI and positron emission tomography (PET) has been established for treatment planning of meningiomas [13–18]. PET-tracers based either on amino acids as [^11^C]-Methionine (MET) and [2-^18^F]-fluoro-L-tyrosine (TYR) or the somatostatin receptor 2 (SSTR2) ligand [^68^Ga]-DOTA-D Phe 1-3Tyr3-Octreotide (DOTATOC) were shown to be beneficial for RT-planning, since these show very high meningioma to background ratios [13–21].

Nevertheless, there are also limitations for MET and DOTATOC: An onsite cyclotron is required to produce MET because of the short physical half-life of 20 min for ^11^C. Due to this fact its use is restricted to a small number of research centers [15, 22]. DOTATOC is easier to handle than MET, but since the pituitary gland also expresses SSTR2 a high uptake of DOTATOC in the sella turcica is physiological and therefore an intrasellar invasion by meningioma cells cannot be differentiated [21]. In such cases, close correlation with CT and MRI is necessary to distinguish meningioma from pituitary tissue.

O-(2-[^18^F]fluoroethyl)-L-tyrosine (FET) is another amino acid-based PET-tracer. In contrast to MET, PET-scans with FET can be performed even by departments distant from a cyclotron, since half-life of ^18^F is more than 5 times longer. Although the uptake mechanisms of MET and FET differ, a close correlation of the intensity of tracer uptake was described in tumoral as well as in non-tumoral cerebral lesions [22, 23]. Moreover, advantageously in comparison to DOTATOC there was no increased uptake in cells of the pituitary gland reported. Therefore, FET was hypothesized to be a superior tracer in the imaging of meningiomas, although the clinical experience of FET-PET in meningioma patients is still limited [24, 25].

Only recently, an EANO taskforce generated a guideline for diagnosis and treatment of meningiomas [26]. However, inter-institutional differences in the use of PET-tracers for the treatment planning process for meningiomas exist. So the aim of this study was to evaluate the influence of these different types of PET-tracers on GTV-definition in meningiomas.

## Methods

### Patients

Between October 2010 and February 2012 21 patients suffering from intracranial meningiomas underwent neuroimaging including CT, MRI, FET- and DOTATOC-PET for treatment planning at the Department of Radiation Oncology in Heidelberg, Germany.

All four examinations were performed in a mean period of 7.7 days (range from 2 to 27 days). The mean time lag of the performances of DOTATOC- and FET-PET-scans was 4.5 days (range from 1 to 21 days).

Nineteen of the 21 patients (90.5%) had undergone surgery for their meningioma in the past and therefore a histological grading could be obtained. Six patients (28.6%) had had even more than one surgical resection in their lifetime. The median interval between the last surgical resection and the performance of the CT-, MRI- and PET-scans evaluated in this study was 45.4 months (range from 3 to 204 months). For patient characteristics see Table 1.

**Table 1:** Patient characteristics

Age	[Years]	
Median age	50	
Range	26 - 75	
Sex	[n]	[%]
Female	16	76
Male	5	24
Main anatomical localisation		
Skull base	17	81
Parasagittal region	3	14
Convexity	1	5
Treatment concept		
RT after incomplete resection	3	14
RT at tumour progression	16	76
Primary RT	2	10
Surgery in past	19	91
Histological subtype		
WHO grade I	12	57
WHO grade II	6	29
WHO grade III	1	5
Unknown	2	10

*RT* radiation therapy

The performance of this study was approved by the local ethics committee (Ethikkommission Medizinische Fakultät Heidelberg).

### Imaging

All patients received an individually formed fixation device including scotch-cast masks for seven patients (33.3%) or thermoplastic masks for 14 patients (66.7%) to immobilize the patient’s head as described before [2, 27].

CT-scans were performed using a Siemens Sensation 4 (Siemens, Erlangen, Germany) with slice-thickness of 3 mm after application of contrast medium (1,5 ml/kg body weight, Ultravist 300, Bayer, Leverkusen, Germany).

MRI was performed in most patients using a 3.0-T MR scanner (Siemens Trio or Siemens Verio). T1-W images were obtained after administration of contrast fluid according to the body weight (Magnograf, Marotrast GmbH, Jena, Germany or Gadovist, Bayer, Leverkusen, Germany) with slice-thickness of 1.3 mm (TR 1.710; TE 4.04). Three patients received T1-W images using a 1.5-T MR scanner (Siemens Symphonie) with slice-thickness of 3 mm (TR 13; TE 4.7) after application of contrast medium (MultiHance, Bracco IMAGING, Konstanz, Germany) since a 3.0-T MR scanner was not eligible or not available in a timely manner.

^68^Ga-DOTATOC was produced as previous published and injected intravenously as bolus [28]. The mean administered activity was 168 MBq (range: 102-197 MBq) and the peptide quantity was 12.5 μg DOTATOC in all preparations. The examination was done with a Biograph-6 PET/CT (Siemens). Approximately 30 min after injection a diagnostic CT of the head was performed (250 mAs, 110 kV, slice collimation 6 × 2 mm, slice-thickness 3 mm Full Width at Half Maximum (FWHM), 0.9 Pitch) which was also used for attenuation correction. One bed position with a 15.5 cm field of view was acquired with 10 min scan time and reconstructed with an ordered subset expectation maximization (OSEM) algorithm with four iterations of 16 subsets and Gauss filtering to achieve images with a matrix size of 256 × 256 and an in-plane spatial resolution of 5 mm FWHM.

^18^F-FET was obtained commercially from IASON (Graz, Austria) and was injected intravenously as bolus with a mean activity of 184 MBq (range: 158-218 MBq). The examination was also done with the Biograph-6 PET/CT. A native low-dose CT (60mAS, 130 kV, slice collimation 6x3mm, slice-thickness 5 mm FWHM, 1.5 Pitch) was done for attenuation correction. The emission was acquired as a dynamic scan and then a static frame covering 20-40 min after injection was reconstructed with the OSEM algorithm (two iterations of 16 subsets, Gauss filtering, matrix size of 256 × 256, in-plane spatial resolution 5 mm FWHM). The uptake of FET and DOTATOC was quantified by standardized uptake values (SUVs).

### Image fusion, GTV delineation and quantitative analysis of tumour volumes

Using Siemens COHERENCE Dosimetrist (Siemens Medical solutions, Concord, CA) image fusion was performed by matching contrast enhanced CT-images with contrast enhanced MRI-T1-W images, FET- and DOTATOC-PET images. Because of a high accuracy of the used image fusion method, errors in co-registration were reduced to a minimum. Therefore, a head fixation device for MRI- and PET-scans was not stringently necessary.

At first tumour extension was delineated by cooperation of two experienced radiooncologists after image-fusion of CT and MRI in each patient, resulting in GTV_CT/MRI_, that included any macroscopic tumour suspicious tissue in contrast enhanced CT and MRI. In a second step in every patient distinct GTVs based on FET- and DOTATOC-PET were contoured (GTV_FET_, GTV_DOTATOC_) by the same advanced radiooncologists in cooperation with an experienced nuclear radiologist respectively. Since a general cut-off value for the SUVs of DOTATOC as well as FET is missing so far, the PET-window levels were adjusted so that the PET-delineation was in accordance with the GTV_CT/MRI_ on slices with a clear differentiation of tumour borders on CT and MRI as described by Thorwarth et al. [18] before.

**Fig. 1:**
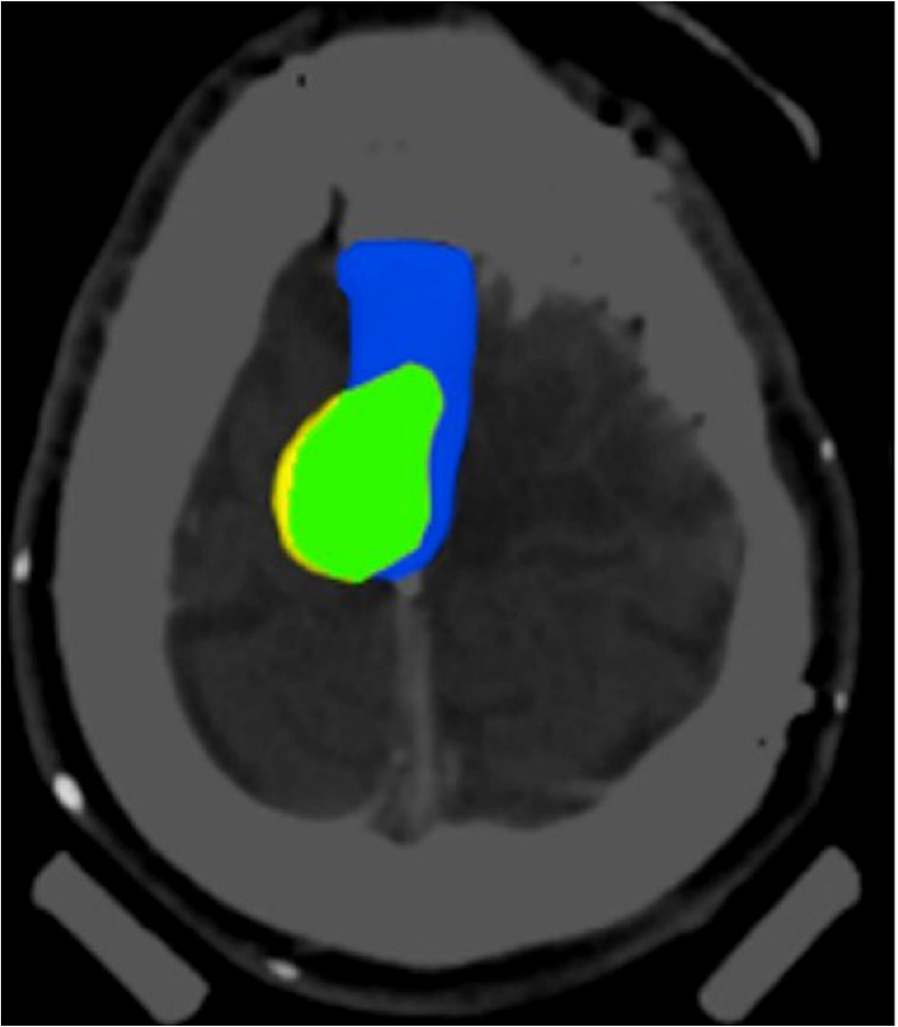
Corresponding volume (green) of GTV_CT/MRI_ (blue) and GTV_FET_ (yellow) of one patient. For more details of this patient including CT-, MRI-, DOTATOC- and FET-PET-images see Fig. 5a

Afterwards GTV_FET_ was compared with GTV_DOTATOC_ as well as GTV_FET_ and GTV_DOTATOC_ were compared with GTV_CT/MRI_. The relative overlap and non-overlap volumes of these GTVs were calculated as described before by Astner et al. [13] (Fig. 1): In detail, the data analysis of each volume using the results of the fusion images was performed. Then, a common volume included the volumes derived from MRI and CT showing PET-uptake. Thereafter, volumes of PET uptake outside the volume GTV_CT/MRI_ was determined and rated as an increase in volume of GTV_CT/MRI_. Lastly, the volume of GTV_CT/MRI_ outside the PET-changes was defined as an increase in GTV_PET_.

All GTVs shown in this study were retrospectively delineated for research only to avoid systemic interpersonal differences since the original 21 GTVs used for treatment planning were contoured by different radiooncologists. The original irradiation plan for patient treatment was calculated with DOTATOC-based GTVs mainly since DOTATOC is the standard tracer in our centre for meningioma radiotherapy planning and there was no large experience with FET-PET in meningiomas so far.

### Statistics

Statistical analyses were performed with IBM SPSS Statistics Version 20 (IBM, Armonk, NY). The relative corresponding volumes were statistically tested using the T-test depending on the type of tracer. The relative corresponding volumes of GTV_DOTATOC_ and GTV_FET_ were tested depending on WHO-grade. All statistical tests were performed at a 5% level of significance.

## Results

In all patients meningiomas could be clearly identified on CT, MRI and DOTATOC-PET. In contrast, the sole anaplastic meningioma showed no enhancement of FET and another patient with two meningiomas of the skull base had one FET-positive and one FET-negative lesion.

**Fig. 2:**
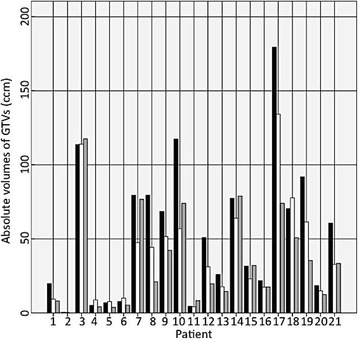
Absolute volumes of GTV_CT/MRI_ (black), GTV_DOTATOC_ (white) and GTV_FET_ (grey) of each patient

The mean volumes of GTV_CT/MRI_, GTV_FET_ and GTV_DOTATOC_ were 53.9 ccm (range 0.46 – 179.5 ccm), 34.7 ccm (range 0 – 117.6 ccm) and 39.4 ccm (0.2 – 134.2 ccm) respectively (Fig. 2). The GTV_DOTATOC_ enclosed a mean minimum relative SUV of 56.1% (standard deviation 0.02) and the GTV_FET_ a mean minimum relative SUV of 65.9% (standard deviation 0.05) respectively. The mean minimal SUV-lesion to normal ratio (LNR) for the delineated GTV_FET_ was 1.5 (standard deviation 0.45).

**Fig. 3:**
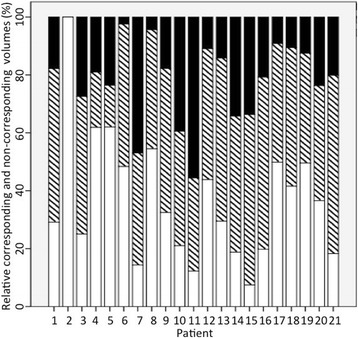
Relative corresponding volumes (striped) of GTV_FET_ and GTV_DOTATOC_ as well as non-corresponding volumes with sole uptake of FET (black) or DOTATOC (white) of each patient

The mean relative corresponding volume of GTV_FET_ and GTV_DOTATOC_ was 41.9% only (range 0 - 61.6%). In WHO Grade I meningiomas there was a mean relative corresponding volume of 45.9% whereas in atypical meningiomas it was less with 39.9% (*p* > 0.05). Since there was no enhancement of FET in the sole anaplastic meningioma, a relative corresponding volume of both PET-CTs could be calculated neither for this patient nor for anaplastic meningiomas (Fig. 3).

There was a significantly stronger correlation between GTV_CT/MRI_ and GTV_DOTATOC_ than between GTV_CT/MRI_ and GTV_FET_ with mean relative corresponding volumes of 52.0% and 36.8%, respectively (*p* < 0.001).

## Discussion

This is the first report of an intraindividual comparison of an amino acid-based PET and DOTATOC- PET in patients with intracranial meningiomas. Furthermore, we present the largest population of patients suffering from intracranial meningiomas that were examined by FET-PET so far.

A trimodal image fusion of CT, MRI and PET is well established in the treatment planning process for RT of intracranial meningiomas. The MRI provides a high accuracy especially in soft tissues. The CT is necessary for accurate computation of radiation doses and helps to estimate the involvement of bony structures. Additional information about tumour extension and biology are presented by PET-scans [15]. The widely used PET tracer ^18^F-fluorodeoxy-glucose (FDG) mostly fails for brain tumour imaging because of the physiologically high metabolism of glucose in the cerebral cortex. In contrast to FDG, the amino acid-based tracers and DOTATOC have been shown to offer high meningioma to background ratios.

Milker-Zabel et al. [16] reported a significant modification of PTV for FSRT in 19 of 26 patients (73%) combining MRT and CT with DOTATOC-PET for treatment planning of meningiomas. Similar results were shown by Gehler et al. [14]. The DOTATOC-PET gave additional information for the GTV-delineation in IMRT-planning in 65% of their cases. By combining MET-PET with CT and MRI Astner et al. [13] demonstrated an influence on GTV in 90.6%. In most cases of their study this resulted in a smaller GTV. By addition of MET-PET they could better discriminate non-tumorous structures as the cavernous sinus, the sella region or meningeal reaction after previous surgery. Furthermore a reduction of the interobserver variability in target volume definition of meningiomas was reported by Grosu et al. [15] using MET-PET for treatment planning. Rutten et al. [17] found discrepancies between MRI and TYR-PET in 46% of intracranial meningiomas in their study. From these cases 83% of the PET lesions extended beyond the MRI lesion. In summary a benefit for additional information regarding the extension of meningiomas was shown for DOTATOC as well as for amino acid-based tracers [16, 17, 21, 29]. Up to now there was a missing evidence supporting the superiority of one of these tracers [14].

FET-PET was shown to be more accurate than FDG-PET for detecting malignant brain lesions [30]. To date experiences for FET-PET in meningiomas are rare [24, 25]. In a PET-comparison study of brain lesions Lau et al. [30] included only one anaplastic meningioma. In other studies comparing FET- and MET-PET of intracranial malignancies, no patient suffering from a meningioma was investigated [22, 23]. Although a close correlation of the intensity of tracer uptake was described for the amino acid-based tracers MET and FET, the uptake mechanism and further intracellular pathways of these two are quite different [22, 23]: MET is mainly transported by the L-transport-system, a bi-directional amino acid carrier, as well as by the A-system and enters several biochemical pathways [17]. It is utilized for protein synthesis, converted to S-adenosyl-methionine e.g. as precursor for polyamine synthesis or metabolised by decarboxylation [23]. As an analogue of tyrosine the uptake of FET is thought to be selectively mediated by LAT2, a L-transporter subtype. Moreover it is not incorporated into proteins or otherwise metabolized [23]. However, although the transport mechanisms of FET seem to be rather specific, the previously reported clinical results concerning neuroimaging seem rather comparable to MET [22, 23].

**Fig. 4:**
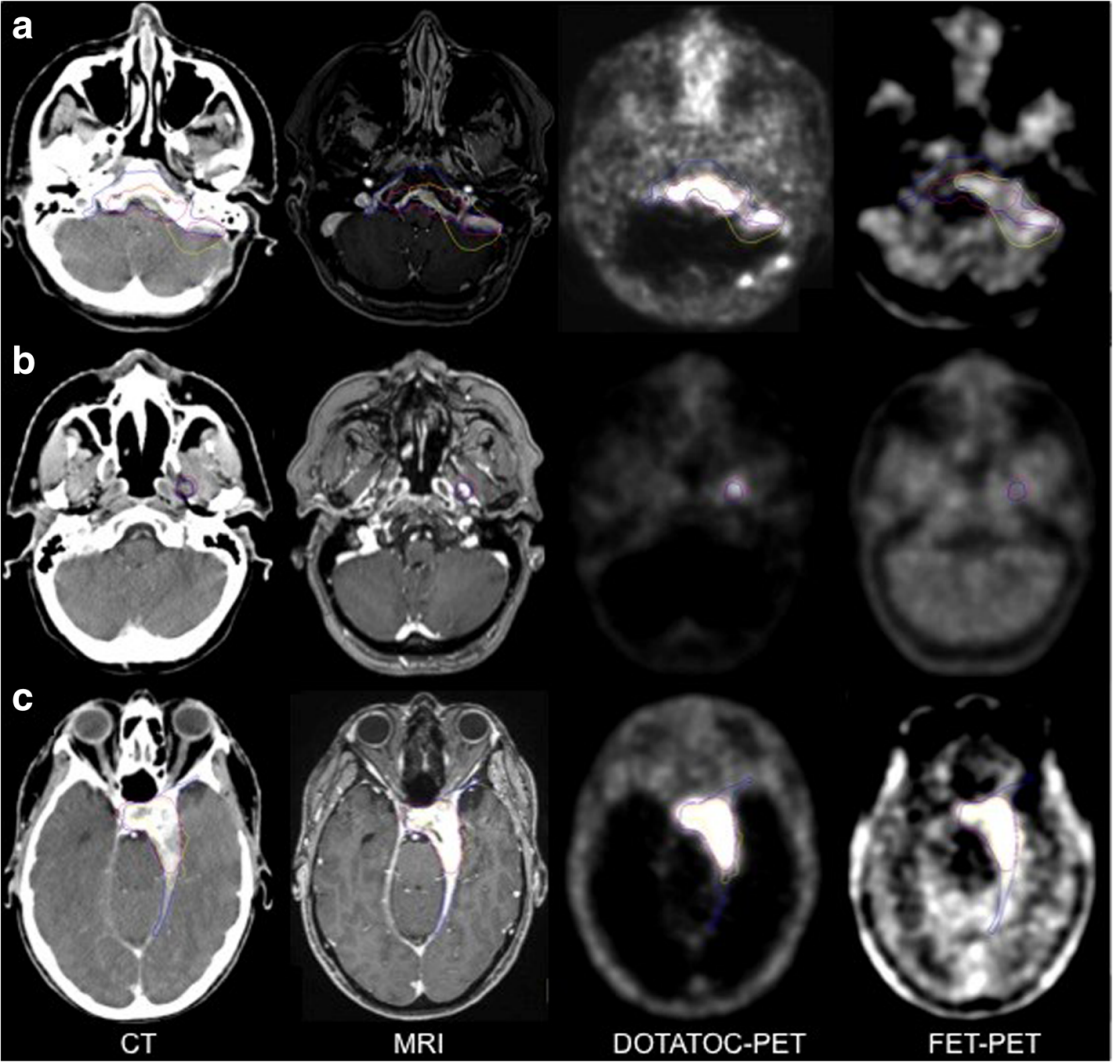
Delineated GTVs of three more patients based on CT and MRI (blue), DOTATOC-PET (purple) or FET-PET (yellow): Meningioma and parts of the neighbouring brain tissue show similar uptake in FET-PET (A + C). A group of three small meningiomas next to the dorsal left cerebellar hemisphere is well visible in MRI and DOTATOC-PET but shows no uptake of FET (**a**). An extracranial branch of the meningioma is not visible in FET-PET since it is neighbouring muscular structures (**b**). The FET-PET clearly shows the complete tumour infiltration of the pituitary gland but also a strong uptake at the sagittal venous sinus and temporal muscles (**c**)

Our data shows a significantly better correlation between CT/MRI and DOTATOC-PET than between CT/MRI and FET-PET. Because of the receptor targeting, DOTATOC is known to be more specific for meningiomas with the known limitation in areas close to the parasellar region since the pituitary gland expresses SSTR2 [21]. In our study, nine of 21 meningiomas were neighbouring this region, so in these patients DOTATOC failed to discriminate between meningioma and pituitary gland. When a clear delineation between meningioma and pituitary gland was not possible we included this organ in the GTV_DOTATOC_ for the evaluation of this study. Compared to DOTATOC, FET is known not to accumulate in the pituitary gland. Despite this advantage, we also have to report about limitations of FET-PET. Due to a low urinary excretion of FET (22% after 5 h), the concentration in blood compartment was found to be relatively high especially in the first hour after injection [31]. Therefore, a visualisation of large vessels as venous sinus can be misinterpreted and the clear delineation of a neighbouring meningioma becomes difficult (Fig. 4c). Moreover, in cases where meningioma cells are neighbouring muscular structures there is more “noise” of the surrounding tissue in FET-PET which results in a reduced discrimination of the meningioma’s branches growing through the holes of the skull base to extra cranial sites [31] (Fig. 4b). In several patients the GTV_n_ exceeded the GTV_DOTATOC_ as well as the GTV_CT/MRT_ by including the neighbouring cerebral cortex (Fig. 4a and c, 5a). It was hypothesized before that an enlarged PET-GTV could be caused by a microscopical infiltration of meningioma cells into surrounding tissues following vascular structures or cranial nerves [17]. Limitations of manual tumour contour delineation or suboptimal windowing of PET-images could be other explanations for slight discrepancies of the different GTV [22]. However compared to DOTATOC we observed a much stronger uptake of FET in the normal cerebral cortex located close by as well as distant from the meningioma. This was also reflected in a mean minimum LNR of FET of 1.5 with a standard deviation of 0.45.

**Fig. 5:**
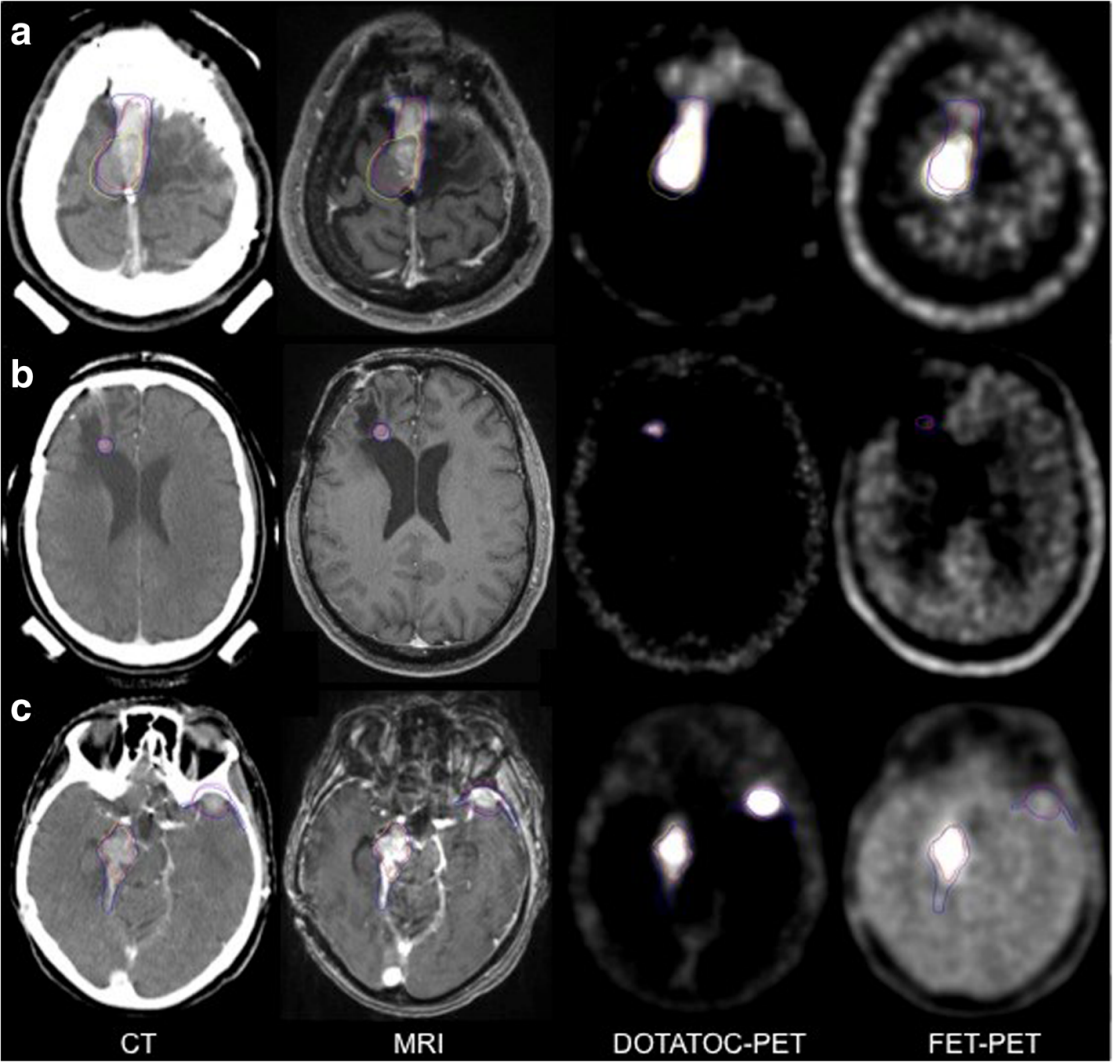
Delineated GTVs of three patients based on CT and MRI (blue), DOTATOC-PET (purple) or FET-PET (yellow): Meningioma is partially FET-negative (**a**), Meningioma is completely FET-negative (**b**). Patient C (**c**) is suffering from two meningiomas, one FET-positive and the other FET-negative. All meningiomas are clearly visible in DOTATOC-PET, CT and MRI

Moreover we have to report about two completely and several partially FET-negative meningiomas showing no FET-uptake in areas highly tumour-suspicious in CT, MRI and DOTATOC-PET (Fig. 5). An explanation could be that amino acid-based tracers are known to provide functional aspects of the activity of meningioma cells and for FET-negative areas maybe a lesser activity up to no activity of growth is to hypothesize. For gliomas it was found that accumulation of MET correlates better with histological tumour spread than CT or MRI [31]. For MET-uptake in meningiomas a significant correlation to Ki-67 index was shown [23] and Gudjonsson et al. [32] could demonstrate that a proton radiation of meningiomas led to a reduction of MET uptake of 19.4%. Potentially in further studies GTV-delineation could be restricted to FET-PET positive parts of meningioma only. But since both FET-negative meningiomas of our study were grown in size in further follow up, this modus operandi seems too hazardous, potentially underestimating the actual extension of the tumour. So apart from the parasellar region DOTATOC seems to be more sensitive and also more specific for the delineation of meningioma tissue than FET.

In comparison to other studies that investigated the effect of amino acid-based tracers in treatment planning of meningiomas we included much more histologically proven high-grade meningiomas: one meningioma grade III and six meningiomas grade II, in total 33.4% (Rutten et al.: 0% (0/11) [17], Grosu et al. 0% (0/10) [15] and Astner et al. 3.1% only (1/32) [13]). This is remarkable since both patients with FET-negative meningiomas in our study were suffering from a high-grade meningioma and the missing FET-uptake could possibly be caused by a different biology in comparison to low-grade meningiomas.

Thus, the data from the present analysis describes differential tracer uptake of DOTATOC and FET in meningiomas as a first intraindividual comparison. To date, the questions whether one or the other tracer is superior cannot definitely be answered. Further investigations are necessary to clarify the minor congruence of DOTATOC- and FET-PET in meningiomas. Histological und molecular biological examinations of bioptic material taken from areas with different tracer uptake could help to understand the reasons. Alternatively, clinical studies could evaluate the outcome after irradiation of the FET-positive tumour parts only. However, as highly tumour suspicious areas remained FET-negative and were grown in size in further follow-up, a restriction to FET-positive areas cannot be recommended in treatment planning of meningiomas so far. Although a close correlation between the uptake of the amino acid-based tracers MET and FET was described in literature, our results of the FET-uptake in meningiomas should not be generalized for other amino acid-based PET-tracers. Despite the known limitations in the area close to the pituitary gland, DOTATOC-PET remains to be our in-house standard, as DOTATOC is known to be more specific for meningiomas and as we have gained a lot of experience with DOTATOC-based treatment planning for irradiation of meningiomas over the last decades [16, 29, 33, 34].

## Conclusion

Volumes based on FET and DOTATOC in meningiomas can be hetereogeneous based on the tracer applied. In general DOTAOTC-PET shows the best overlap with MR/CT. Thus, based on the data from this analysis in concordance with published data DOTATOC-PET is recommended for treatment planning of meningiomas and should be implemented when available.

## Abbreviations

CT: Computed tomography
FDG: ^18^F-fluorodeoxy-glucose
FET: O-(2-[^18^F]fluoroethyl)-L-tyrosine
FWHM: Full Width at Half Maximum
GTV: Gross tumour volume
MET: Methionine
MRI: Magnetic resonance imaging
OSEM: Ordered subset expectation maximization
PET: Positron emission tomography
RT: Radiation therapy
SSTR2: Somatostatin receptor 2
SUV: Standardized uptake values
TYR: Tyrosine
DOTATOC: [^68^Ga]-DOTA-D Phe 1-3Tyr3-Octreotide
